# Sensor and Display Human Factors Based Design Constraints for Head Mounted and Tele-Operation Systems

**DOI:** 10.3390/s110201589

**Published:** 2011-01-27

**Authors:** Andre Harrison, Linda Mullins, Ralph Etienne-Cummings

**Affiliations:** 1 Department of Electrical and Computer Engineering, Johns Hopkins University, 105 Barton Hall 3400 N, Charles St. Baltimore, MA 21218, USA; E-Mail: retienne@jhu.edu; 2 Human Research and Engineering Directorate, Soldier Performance Division, Visual and Auditory Processes Branch, Spatial Perception Research Team, RDRL-HRS-S Aberdeen Proving Ground, MD 21005, USA; E-Mail: lmullins@arl.army.mil

**Keywords:** high dynamic range, tone mapping, sensor design, human factors, head mounted, mobile sensor, tele-operation, human visual system

## Abstract

For mobile imaging systems in head mounted displays and tele-operation systems it is important to maximize the amount of visual information transmitted to the human visual system without exceeding its input capacity. This paper aims to describe the design constraints on the imager and display systems of head mounted devices and tele-operated systems based upon the capabilities of the human visual system. We also present the experimental results of methods to improve the amount of visual information conveyed to a user when trying to display a high dynamic range image on a low dynamic range display.

## 1. Introduction

Imagers are commonly designed with the goal of producing a system with the lowest power, widest dynamic range, fastest frame rates, *etc.* However with frame rates of >10,000 fps [1,2] and dynamic ranges >160 dB [3–5], one must ask what are the best capabilities one can desire in an imager? In general it is hard to answer such an open ended question as it is highly dependent on the application of the imager, but if we restrict our focus to imaging systems where the output is viewed by a person in real-time, one can determine specific constraints for the design of the sensor based on the capabilities of the human visual system (HVS) [6,7]. It is also important to know what the capabilities of the display are as it is the only way to present the information captured by the sensor to the user and any limitations in it may adversely affect the amount of information that can be presented to the viewer. This limits our intended scope of imagers to night vision goggles, infrared imagers, and other multi-spectral imagers used by people, tele-operation systems such as remote controlled bomb disposal units, UAV’s, and cars with night vision HUD or rear view cameras. This paper also has application to some extent any sort of head mounted device even ones where the camera exists solely in software and the displayed scenes are virtual.

There have been other papers that discuss the physiological properties of the HVS and how they may affect the design of a sensor or a display, but most of these papers talk very little about the specifics about the resulting constraints on the sensor or the display and spend most of the document describing how the HVS works. In this paper we determine the most desirable sensor and display system in terms of describing a system that maximizes the amount of visual information captured and sent to the user. To do this we focus on sensors and displays that match the perceptual capabilities of the HVS using data gathered from psychophysical studies.

The rest of this paper is broken into two sections first we describe different sensor properties that are affected by the perceptual limitations of the HVS and what the best sensor system should be in order to maximize the amount of visual information captured that could be shown to the viewer. For this section we assume a display that can show all the information the sensor captures. The second half of this paper focuses on the necessary capabilities of a display to ensure that is able to show all the visual information the sensor captures. We also describe work we have done to maximize the amount of visual information shown to a viewer when the dynamic range of the display is much lower than the dynamic range of the visual information.

## 2. Sensor Constraints

The goal, as we see it, of image sensors used in tele-operation and head mounted devices is to try and capture the same amount of visual information as a persons can capture from a scene. The simplest way to do this is to match the capabilities of the sensor with that of the H*VS.* To this end we describe the perceptual limits and physical properties where relevant, of the H*VS.*

### 2.1. Spatial Resolution

Contrast sensitivity tests show black and white gratings of varying intensity and spatial frequency in order to determine the highest spatial frequency where a person can still perceive changes in shading for different contrast levels. From these experiments contrast sensitivity curves have been created along with models of the contrast sensitivity of an average person. From this we can say that the highest spatial frequency a person can perceive is 60 cycles/degree regardless of the change in contrast [6,8,9]. This then suggests that sensors should have a maximum resolution of ∼14,400 × 24,000 pixels, if the entire FOV of the HVS is covered [10].

**Figure 1.**
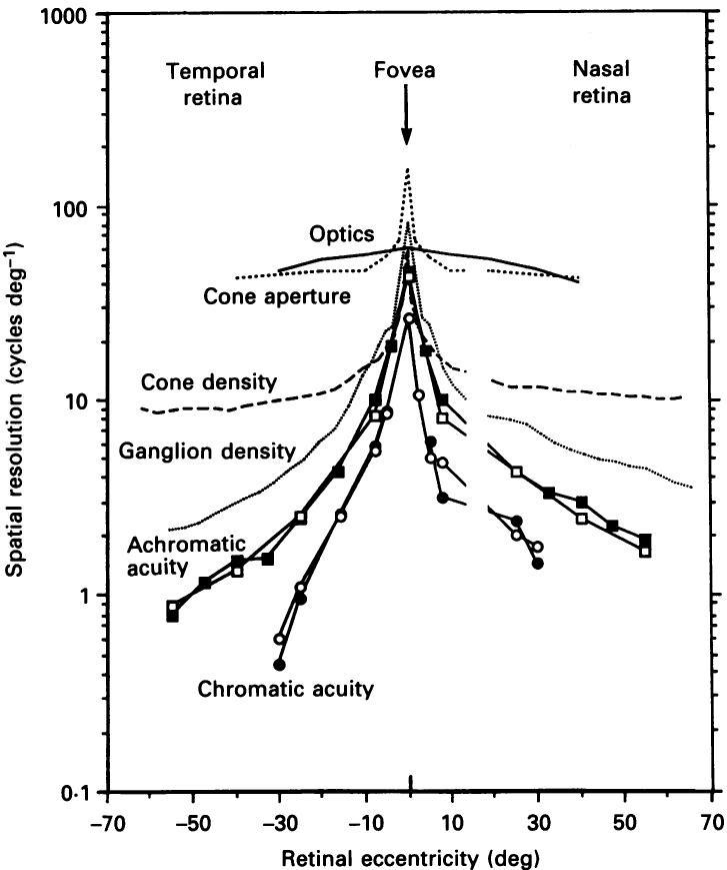
Psychophysical data for the human eye and Optical and Retinal limits of vision. Psychophysical data obtained using drifting gratings at various spatial frequencies, but for a fixed temporal frequency, 8 Hz. Reproduced with permission from [9].

It is often noted that the spatial resolution of the eye is not uniform throughout the retina. Near the center of the eye in the fovea the spatial resolution of the eye is highest and that resolution drops off quickly as eccentricity increases, at a rate of approximately 1/x as shown in Figure 1. A person is able to view a scene as if it is all viewed at the highest resolution everywhere by quickly moving their fovea over the entire visible area. This means that a sensor that has a pixel density of 120 pixels/degree is actually capturing more information than the average HVS is able to. Such a sensor system can reduce the amount of information it must capture and then transmit by using a foveated design [10–12]. The pixel density at the periphery of a future foveated sensor system could be 1–2 orders of magnitude lower than the pixel density at the center of the sensor system. This would reduce the total number of pixels and the overall amount of information that needs to be transmitted. However the overall system would be come more complex as it would then require a way to move the fovea of the sensor over the visible area and an eye-tracking system in order to sync the motion of the viewer’s eye with the movement of the sensor over the visible area.

### 2.2. Temporal Resolution

**Figure 2.**
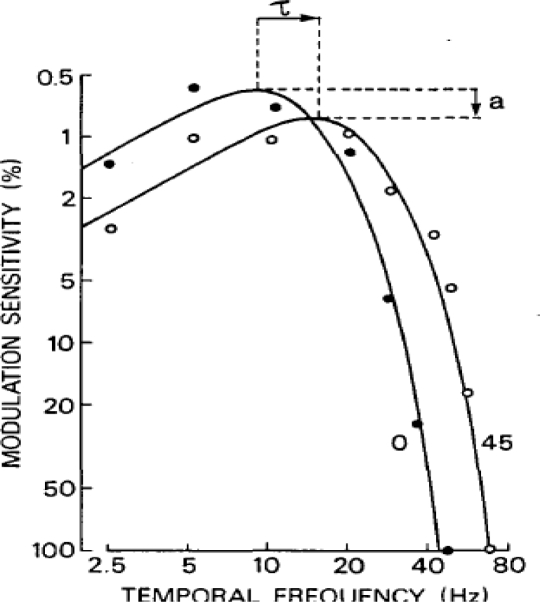
Temporal sensitivity for two different eccentricities (0 and 45 degrees) Reproduced with permission from [17].

There have been numerous experiments conducted on the human eye to try and measure and characterize the temporal sensitivity of the H*VS.* These experiments show that the sensitivity of the eye is highly dependent on the size, speed, brightness, and location of the visual stimulus [6,13–16]. However for the design of an arbitrary image sensor matched to the perceptual capabilities of the HVS the most important temporal parameter is simply what is the highest temporal stimulus a person can perceive. This is captured from critical flicker frequency experiments, which present two impulses of light to the eye separated by a small period of time. The shortest delay between impulses that a person can detect is then one over the critical frequency. Figure 2 shows the profile of temporal sensitivity of a person with approximately average temporal sensitivity. From the CFF data the maximum frequency the average person can detect is <80 Hz. This then imposes a maximum frame rate on any image sensor and says that past 80 Hz a person will be unable to detect any visual stimulus change. This constraint is a very simple one in terms of existing sensors. Most sensor systems shoot for a minimum of 30 fps already as standard and the fastest sensors can operate many orders of magnitude faster than this [1,2]. So existing sensor systems only need to operate at a rate 2–3 times faster than they already do to be matched to the temporal capabilities of the H*VS.*

### 2.3. Dynamic Range

**Figure 3.**
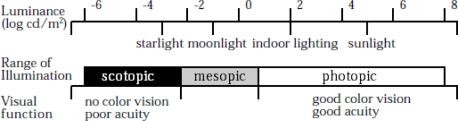
Dynamic Range of the Visual System Adapted from [18].

The HVS is highly adaptable to changes in illumination level; the difference between the darkest stimulus the HVS can detect and the brightest one, given enough time to adapt to these different stimulus levels can span over 12 orders of magnitude, 240 dB, as shown in Figure 3. The HVS cannot detect visual stimuli at these illumination levels at the same time however. The HVS may require a few seconds to over a half an hour to adapt to changes in the average illumination level and detect the desired stimuli. This is akin to the ability of most image sensors to adapt to the average illumination by changing their exposure time, aperture size, and system gain. For a standard off the shelf camera exposure times can change by over 7 orders of magnitude, while state of the art imagers have reported changes up to 10 orders of magnitude, which is close but still well below the capabilities of the HVS [3–5]. The ability of imagers to adapt to illumination level to match that of the HVS is important however what is at least equally as important for matching the capabilities of the HVS is to have the same in scene dynamic range as the average person.

**Figure 4.**
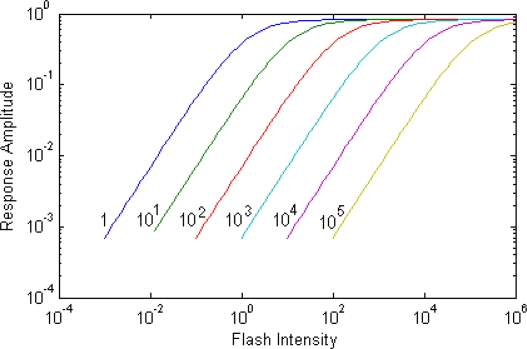
Model of Response of photoreceptor at different adaptation levels. Adapted from [6].

A person’s in-scene dynamic range is the ratio between the brightest and darkest stimuli a person can detect at a given adaptation level. Models of adaptation in the photoreceptor, which agree well with electrophysiological data, show that at a given adaptation level the photoreceptor responds to changes in illumination spanning up to 70 dB as shown in Figure 4 [6]. These models generally have an ‘S’ like and can be described using the Michaelis-Menten equation [19]. This suggests that an imager sensor needs to not only be able to adapt to illuminations by over 12 orders of magnitude, but that it must be able to detect illumination levels that differs by over 70 dB within the same scene. A number of imagers report dynamic ranges of 160 dB or higher [3–5]; however it is often unclear if this is for a specific exposure or adaptation setting representing the full dynamic range or if this is the in-scene dynamic range. Many biologically inspired imagers that use time to first spike and spike rate encoding have no real sensor level exposure control and so their full dynamic range is also their in-scene dynamic range, which is often greater than the in-scene dynamic range of the HVS, but less than the full dynamic range of the H*VS.*

## 3. Display Constraints

The ideal display for a sensor is one that can show all of the visual information that the image sensor has captured and does not exceed those capabilities. Most of the properties of that display then will simply have the same characteristics as that of the sensor. For instance the temporal resolution of the display should match that of the sensor also the spatial resolution should equal the maximum resolution of the sensor. In fact the resolution of the sensor should be set to match the resolution of the display, while the resolution of the display should be set to the maximum resolving power of a person with normal eye sight at the expected viewing distance. For an LCD screen in a tele-operation system where a person is 12–18 inches away from the screen the maximum display density should be 22 pixels/mm, while for a HMD such as NVG or infrared imager the display is usually only 25 mm away and the display, and thereby sensor pixel density should be 126 pixels/mm to in order to present as much visual information as possible. However, there are also several parameters that are relevant to a display that simply are not explicitly part of the sensor design such as color and field of view, which are addressed below.

### 3.1. FOV

The resolution of HDTV screens report highest resolution of 1,920 × 1,080 pixels, while the latest LCD screens have resolutions of 2,560 × 1,600 pixels. If a person were to view either of these screens at a distance where a pixel occupied ∼1/60°, the standard measure of the resolving power of the human eye, the screens would only cover a region 29° × 17° for the HDTV and 37° × 30° for the LCD screen. This is far below the field of view of the human eye and below the recommended viewing areas for tasks often employed while using tele-operated systems or HMD’s such as interacting with the environment and estimating relative motion, though the LCD is close to the 40° FOV recommended for target identification tasks [20,21].

The human eye, while fixated on a specific location, can see an area covering 160° horizontally and 130° vertically. If the eye does not remain fixed, but the head does it can cover an angle of 200° horizontally and 130° vertically. It may be a long time before sensors and displays can cover the entire visual area that the eye can see at the maximum spatial resolution, but many tasks can be accomplished with smaller viewing areas with negligible loss in performance time. There have been many psychological and psychophysical tasks geared at understanding how much area of the environment people need to see in order to perform different tasks and research has found that in general for most visual acuity levels a wider field of view is preferred over an increase in visual acuity. It has not been shown exactly where optimum balance between visual field and visual acuity are, but based on the studies that have been done the optimum field of view is between 70–150 degrees, with 70–90 degrees recommended as a good default setting, this is tempered by the fact that a visual acuity of at least 20/200–20/80 has been shown to be sufficient for most tasks, though 20/40 is required for some driving tasks [20].

### 3.2. Color

Most displays can show millions to billions of different color, but at how many colors can a person actually detect? First a few brief words about color. Within the retina there are two types of photosensitive cells, cones and rods. Rods are monochromatic cells, since they are unable to distinguish between wavelengths of light. Cones on the other hand are color sensitive cells, though they are sensitive only to illumination levels in the mesopic and photopic ranges, (10^−3^cd/m^2^–10^1^cd/m^2^), (10^1^cd/m^2^–10^8^cd/m^2^) respectively, while rods are sensitive to illumination levels from (10^−6^cd/m^2^–10^2^cd/m^2^) [6]. There are three types of cones in the retina long, medium, and short wavelength cones. Each type of cone is maximally sensitive to certain wavelengths, ∼572 nm (red), ∼539 nm (green), and ∼430 nm (blue), respectively though the drop in responsitivity at other wavelengths is broad enough that the curves for the three types of cones overlap for many wavelengths.

**Figure 5.**
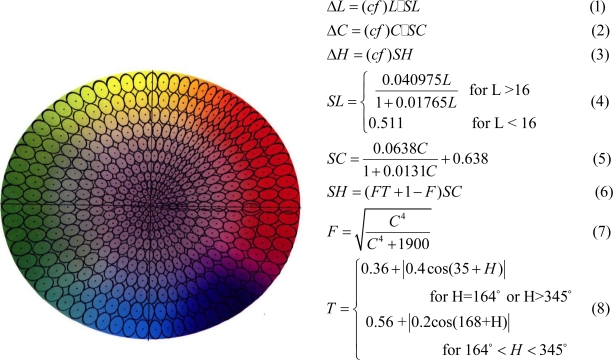
Color Measuring Committee Tolerance chart.

Overall the photoreceptors in the human eye are sensitive to a narrow band of wavelengths within the electromagnetic spectrum, namely 400 nm to 700 nm. The eye interprets what wavelength of light it is seeing based on the magnitude of the response of the long, medium, and short wavelength cones. Display and sensors both generally present and capture, respectively, a combination of red, green, and blue light. However the RGB color space is not perceptually uniform especially since the eyes are not equally sensitive to red green and blue light. In order to present a more linear color space, with respect to the color sensitivity of the human eye, the CIE (Commission Internationale de l’Eclairage*)* developed the CIELAB and CIELCH charts using these charts a color is specified by 3 variables, L*(luminance), a*(redness-greenness), b*(blueness-yellowness) or L*, Chroma, and hue. For a given color the region of color space that appears to be the same color using a JND test is approximately in the shape of an ellipsoid. The Color Measurement Committee, created a tolerance chart that specifies the region around a given color in which all the colors around it appear to be the same. The CMC tolerance chart operates on the CIELCH color space and gives a ΔL, ΔC, and ΔH value for a given L, C, and H value. This defines an area in which the color is perceptually no different than the L, C, and H color. This then allows the CIELCH color space to be divided into small ellipsoids. The total number of ellipsoids in the entire color space is estimated to be over 7 million, Figure 5.

### 3.3. Dynamic Range

It will eventually be possible to create displays that have a dynamic range equal to that of the full dynamic range of the HVS, however for a standard image the dynamic range of that image is only going to span the in-scene dynamic range of the sensor, which should match the in-scene dynamic range of the HVS of ∼3.5 log units or 4 orders of magnitude as shown in Figure 4. If the display dynamic range is much higher than this then visual information may be presented to the user that they cannot detect because that region of the image is too dark or too bright for the current adaptation level of the eye. Most displays however have dynamic ranges well below the in-scene dynamic range of the HVS, and so are unable to display all of the high dynamic range information captured by the sensor. The HVS however is not overly sensitive to absolute levels of illumination, but it detects relative changes in brightness or contrast. Thus it is possible to show most of the visual information from a high dynamic range image in a low dynamic range version using dynamic range compression algorithms.

## 4. Dynamic Range Compression through Tone Mapping

The problem of trying to display an image that has a dynamic range greater than that of the display is not a new one and has been an active area of research in the computer graphics and image processing fields for several years. The research that has been conducted has been mainly concerned with compressing a HDR image and displaying it on a low dynamic range display, essentially trying to convert a 32 bit image or higher into an 8 or 10 bit one, while trying to make the displayed image appear perceptually similar to the HDR image and/or aesthetically pleasing. The various compression methods that have been developed are referred to generally as tone mapping algorithms [19]. However a perceptually similar image does not necessarily maximize the amount of visual information shown to the user. Because of this potential difference we wanted to develop a tone mapping algorithm that actually maximized the amount of visual information displayed to the viewer. To get an idea of what types of tone mapping algorithms work best for this goal we ran a psychophysical experiment comparing well known tone mapping algorithms. These algorithms were chosen based on their popularity, their potential to be implemented in a mobile vision system, and to ensure a representative sampling of the different types of tone mapping algorithms. Based on the results of the first experiment, described below, we decided to modify the bilateral filter tone mapping algorithm, one of the algorithms compared in the experiment, to try and improve the amount of visual information it showed. In the second psychophysical experiment our modified algorithm was tested against the top four performing algorithms from the first experiment to determine if it actually did improve the amount of visual information shown. The following sections describe the first and second experimental procedures, results, and a discussion of those results.

### 4.1. Psychophysical Study

Tone mapping algorithms have historically been designed to create images perceptually similar to their HDR source and/or aesthetically pleasing; which does not necessarily maximize the amount of visual information shown, but as a starting point we decided to see how well they actually did achieve this new metric anyway. The tone mapping algorithms that were included in the study were the Log Adaptation algorithm by Drago [22], the Histogram Adjustment algorithm by Ward [23], the Retinex algorithm by Jobson [24], the Photographic tone mapping operator by Reinhard [25], and the Bilateral Filter algorithm by Durand and Dorsey [26]. These algorithms were chosen as a representative sampling of the field of tone mapping algorithms [19]. They were also selected because of their low computational complexity, in comparison to others of their type, and their susceptibility to creating visual artifacts. Beyond just understanding, which types of tone mapping algorithms presented the most amount of visual information we also intended to use the features of the algorithms that performed the best in the first experiment to design a new tone mapping algorithm that further increased the amount of visual information presented to the user. This meant that the algorithms we choose for the first experiment also had to meet the constraints of the algorithm we wanted to develop. Namely, that it presented as much visual information and was of low computational complexity, so that it could be implemented in a future mobile vision system. To this end two of the algorithms we chose belonged to the simplest class of tone mapping algorithms, known as global tone mapping algorithms. Global tone mapping algorithms are algorithms that directly mapping the value of a pixel in a HDR image to the value of a pixel in the compressed version. This mapping is independent of the pixels location in the image. The two algorithms we used were the log adaptation and histogram adjustment tone mapping algorithms. The other class of tone mapping algorithms that exist are known as local tone mapping operators. Local tone mapping operators use the local illumination and/or gradient information around each pixel in the HDR image to determine the value of that pixel in the compressed image. Local tone mapping operators are usually significantly more computationally complex versus global tone mapping algorithms, but they are often more adaptable to different types of images and generally produce better results. The three local tone mapping operators we used for experiment 1 were the Retinex, the photographic tone mapping operator, and the bilateral filter.

### 4.2. Study Setup and Procedure

**Figure 6.**
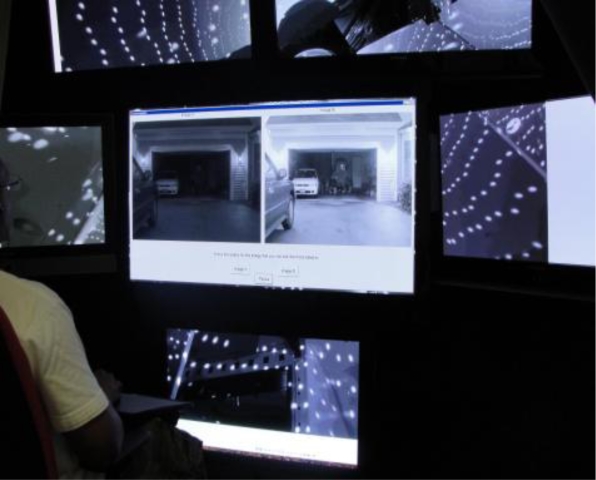
A Picture of the Visual Perception Lab.

In order to conduct the psychophysical testing, using the various image processing algorithms we developed, we needed an experimentation room to run these experiments. To do this we designed and built a visual perception lab, Figure 6, which houses multiple HDTV’s on which different types of visual information can be presented. The room has been designed so that only the visual stimuli during an experiment comes from the TV’s, and reflections are reduced by covering the walls and ceiling in low matte black foam material and carpeting the floor. The image of the Visual Perception lab in Figure 6 shows that the visual stimuli can be displayed on one or more of six HDT*Vs.* The central screens cover the 40° central horizontal axis and 60° central vertical axis of the subject’s field of view while the two peripheral screens are mounted on a swiveling axis to increase the sense of immersion of the subject and increase the horizontal FOV to 97°.

We conducted both experiments in the Perception Laboratory that we designed, but for these experiments we only needed 3 of the available 6 monitors. All of the monitors that weren’t involved in the test were kept off during testing. Participants sat in an adjustable chair, approximately 70–75 inches from the screen. For both experiments five tone mapping algorithms were compared and contrasted. We used ten HDR images for each experiment of different scenes and ran them through the five algorithms to generate fifty test images, which we used in the different experiments. Each experiment consisted of two parts an object detection task, as an objective measure of each algorithm, and a paired comparison task, as a subjective measure.

### 4.3. Experiment 1

**Figure 7.**
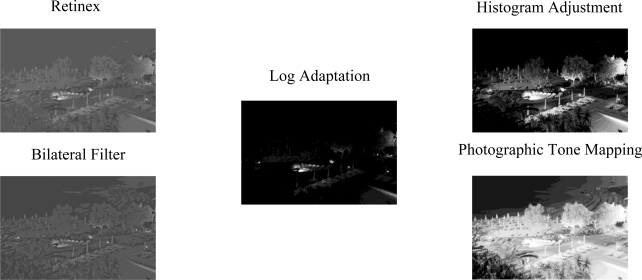
Example images generated using the different tone mapping algorithms.

For the first experiment, thirty students and individuals from communities around The Johns Hopkins University were used as test participants. Participants were between the ages of 18 and 35, had a minimum visual acuity of 20/30. Their vision was tested using an OPTEC® 5000 vision tester and were paid $20.00 per hour for their participation. They were also asked to fill out a standard demographic questionnaire. For the first experiment the following tone mapping algorithms were used to generate the test images. The Log Adaptation algorithm by Drago [22], the Histogram Adjustment algorithm by Ward [23], the Retinex algorithm by Jobson [24], the Photographic tone mapping operator by Reinhard [25], and the Bilateral Filter algorithm by Durand and Dorsey [26] Figure 7.

**Figure 8.**
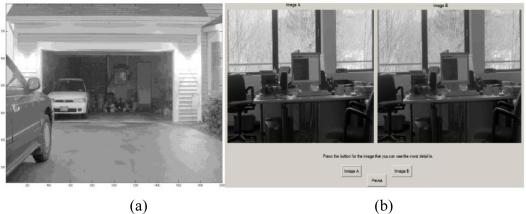
Visual Perception Study Images. **(a)** An example image from the target detection task. **(b)** Is an example image of the paired comparison task.

For experiment 1 the first task was the objection detection task Figure 8(a). For this task, one image was presented at eye level on the center monitor of the perception lab. By the end this session each participant viewed 10 of the 50 images—1 image generated from each of the 10 HDR images; he/she did not see the same scene more than once. The choice in images used was controlled so that every algorithm had generated two of the images shown. Each image was displayed for 60 seconds and the participant’s task during that time was to identify as many objects as possible in as much detail as they could. The list of objects the participant could identify was open-ended; meaning any item in the scene was potentially an object. Also the participants were asked to identify regions that appeared to have no objects, featureless sections. The thinking behind this was algorithms that displayed more detail than others would allow participants to identify more objects or would allow them to identify objects in greater detail than others. Conversely lower performing algorithms would have more instances of blank or featureless regions.

Participants used a computer mouse to move a cursor so that it pointed at the object they were identifying while verbally saying the name of the object. Each test session was also video recorded for later analysis. The video recorder was placed behind the observer, so that the screen image and the mouse cursor were visible on the recording, but the participants face was not recorded. The number of objects correctly identified was determined from viewing the video tapes after testing. Participants did not receive feedback on their performance during testing. A correctly identified object was defined as any object that was selected using the mouse and verbally identified by a statement that accurately described the selected object.

The second task was a paired comparison task Figure 8(b). For this paired comparison task two images, of the same scene, but generated using different tone mapping algorithms, were presented side by side at eye level. Both images were presented on the center monitor. Images were displayed as a split image on a single monitor so that video settings were consistent across the two images being compared. Each participant had to make 100 comparisons to complete this task. Every algorithm was paired with every other algorithm for a given scene, totaling 10 comparisons per scene. The 10 scenes and 10 comparisons per scene resulted in 100 comparisons. For the pairs of images shown the decision to show one image on the right or the left of the TV was randomized. Participants were asked to compare pairs of images and select the image they believed had the most amount of visible detail. For this task we created a graphical user interface in MATLAB to present the images and allow the participants to make a choice between the two images. This served as the only record of the participant’s task, there was no video recording. Upon selection the choice made and the time taken to make that selection were recorded for later analysis. This task was a forced choice, the participant had to select either the left image or the right image and he/she was allowed to take as much time as needed to make that selection, Participants were instructed to try to limit their selection time to one minute or less, but this was only a suggestion. Selection times exceeding one minute were possible and did occur occasionally.

#### 4.3.1. Experiment 1 Results and Discussion

The dependent measure for the object detection task was the percentage of correctly identified targets. For each image, the total number of possible targets was determined by totaling the number of distinct objects correctly identified across all participants, regardless of the algorithm. This number was used as the maximum number of possible targets for a given image. The percentage of correctly identified targets was calculated using the number of correctly identified objects for an individual participant and the maximum number of targets for the corresponding image. A mixed model analysis of variance indicated there were not any statistically significant differences between algorithms for the object detection task. This may have happened because participants would identify the most obvious objects first. These objects also happened to be the objects that showed up across algorithms. Participants rarely identified objects that appeared when run through one algorithm, but not the other.

**Figure 9.**
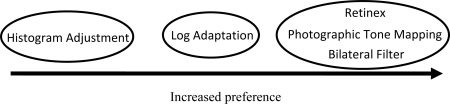
Diagram summarizing the paired comparison results of the first psychophysical experiment. Algorithms are listed from left to right in order of increased preference; in terms of how much visual information or detail seemed to be visible by test subjects.

There were significant differences in the paired comparison task however, the results of which are tabulated in Table 1. For the paired comparison task the results were collapsed across images to determine whether one algorithm was preferred over another regardless of the scene that was being viewed. This resulted in 300 comparisons between each pair of algorithms and showed that the bilateral filter, Retinex, and photographic tone mapping algorithms were the most preferred. There was not a statistically significant difference between the Retinex, bilateral filter, and photographic tone mapping algorithms Figure 9. The log adaptation algorithm was preferred less than the bilateral filter, Retinex, and photographic tone mapping algorithms. The histogram adjustment algorithm was the least preferred in terms of amount of detail that appeared in the final image.

### 4.4. Automated Bilateral Filter

From the results of the first experiment we knew that our new tone mapping algorithm would be a local one, however the results did not shed any light on what type of spatial filter our new algorithm should be based on. Almost all local tone mapping operators use some type of spatial filter to estimate the local features in an image. Since there appeared to be no perceptual differences we looked at the computational differences between each algorithm. The Retinex tone mapping operator runs the HDR image through three spatial filters, each at a different scale. Unfortunately, the optimal spatial filter sizes varies based on image size and the spatial properties of the image, but there is no known a priori method to determine what the spatial filter size should be. So an image may need to be filtered at many different scales in order to find the proper three spatial filters. The Photographic tone mapping operator, unlike the Retinex operator adapts to the spatial properties of each image by iterating through a set of center-surround spatial filters of various sizes until an appropriate one is found. This produces consistent results without much human intervention, but can take multiple spatial filters may be tried before the correct one is found. The bilateral filtering algorithm on the other hand uses only a single filter for the entire image. The filter is an edge preserving spatial filter and so effectively adjusts its size based on the spatial contrast properties of in the image, without iterating. The draw back of using an edge preserving filter is that it is significantly more computationally intensive compared to the spatial filters used in the other algorithms. However, Durand and Dorsey came up with a fast way to compute an approximate edge preserving spatial filter that is no more computational complex than any regular spatial filter [26]. Their method also allows the original HDR image to be sub-sampled up to a factor of 20, which significantly reduces the computational cost of algorithm. For these reasons we selected to improve and automate the bilateral filtering algorithm.

A drawback of the bilateral filtering algorithm is that depending on the spatial and illumination properties of the HDR image the final image may come out too dark or too bright leaving large regions without any detail. Whether the image is too bright or too dark is largely dependent on the scaling factor, w, that is used when combining the detail layer with the illumination layer. A simple solution then is to choose a default value for w, and then simply linearly scale the pixel values of the resulting image so that the brightest pixel is set to the highest illumination value, and the darkest pixel to the darkest displayable value. Unfortunately this also often results in sub-par images where images appear to be washed out due to a few extremely bright or dark pixels. This is often the case when the illumination source, such as a light bulb or the sun itself, appears in the image. The idea behind the automation, comes from the fact that some of the above tone mapping algorithms, such as the histogram adjustment and photographic tone mapping algorithm, make pictures look better by sacrificing some of the visibility of the pixels in the images. Instead of trying to ensure that every pixel is visible the algorithm tries to ensure most of the pixels of the detail layer are visible. This is done by controlling the value of the scaling factor, w, such that the average pixel value of the resulting image L_d_ is in the middle of the range of displayable illumination levels (by default we assume that to be 0.5 or 128).

$$\begin{array}{c} J_{s}=\frac{1}{k(s)}\sum_{p\in \Omega }f(p-s)\cdot g\left(I_{p}-I_{s}\right)\cdot I_{p}\\ \text{and}\;k(s)\;=\;\sum_{p\in \Omega }f(p-s)\cdot g\left(I_{p}-I_{s}\right)\\ L_{d}\;(x,y)=\text{exp}\;\left(\text{log}(I(x,y))-\text{log}(J(x,y))+w\cdot \text{log}(J(x,y))\right) \end{array}$$

choose w such that *mean* (*L_d_*(*x*, *y*)) = 0.5

Fundamental Equation of the Bilateral Filter J_s_ is the base illumination of pixel s = (x,y), f and g are edge stopping functions like a Gaussian or Lorentzian filter function, and w is a scale factor used to compress the base illumination layer. The value of L_d_ is described in terms of pixel values.

### 4.5. Experiment 2

The procedure for the second experiment was very similar to the first, but had the following differences. For the object detection task the number of participants was increased to 60 to try increase the weight of the statistics per algorithm, but we kept the number of participants for the paired comparison task to 30. We also changed the payment of participants to a flat rate of $25 for participants who did both the object detection and paired comparison task, and $15 for the participants who only did the object detection task. We also changed the procedure of the object detection task by creating a list of ten objects for each scene that participants had to identify. Each scene had its own list of ten objects and the participants had 30 seconds to try and identify all the targets on the list. The lists were created by choosing targets that appeared to show up well for some algorithms, but not for others.

**Figure 10.**
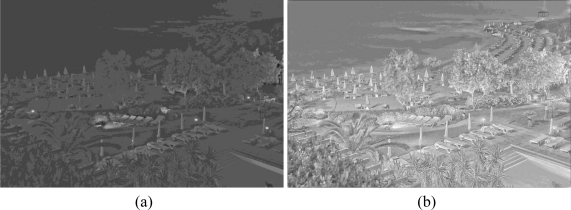
**(a)** Image generated using the bilateral filtering tone mapping algorithm. **(b)** Image generated using the automated version of the bilateral filter tone mapping algorithm to increase amount of visual information seen by the viewer.

For the second experiment we kept the four algorithms from the previous experiment that performed the best, the Retinex, photographic tone mapping, the bilateral filter, and the logarithmic adaption. We also used the modified bilateral filtering algorithm that we developed as the fifth algorithm. The apparent improvement, in terms of amount of visible detail, between the standard bilateral filter and the modified bilateral filter is shown in Figure 10.

#### 4.5.1. Experiment 2 Results

Unfortunately, again results from the object detection task have yet to yield statistically significant results, so we have yet to show that there are any objective differences between these five algorithms. It is unclear whether the objects used for identification simply weren’t sufficient to indicate the differences between the algorithms or that we still had an insufficient number of people to yield any significant results. The results of the paired comparison however, bore significant results. A summary of the results are shown below, but the most important fact is that the participants preferred the new algorithm over any of the other algorithms that were tested, which indicates that the automated bilateral filtering algorithm appears to show more detail than any of the other tone mapping algorithms. Also, again the global tone mapping algorithm, the logarithmic adaption algorithm, was the least preferred in terms of detail.

## 5. Conclusion

The HVS is a highly sensitive, adaptable, and sophisticated sensory system and as the capabilities of imagers and displays improve we must be aware of the possibility of overdesigning the sensor or display system. The ideal mobile vision system is one that captures and presents as much information to the user as they can process without exceeding those limits. This says that the sensor and display system should have similar spatial resolution, temporal resolution, dynamic range, *etc.* as the average user’s eyes. We must thereby understand what these capabilities are in order to optimally design imager systems intended for human use. Also, by understanding not only the capabilities, but the underlying physiology a simpler system can be designed with negligible effective differences. The basic example being that the entire visible spectrum can be reproduced using only three wavelengths of light, red, green, and blue, at least as far as the HVS is concerned. Understanding the underlying physiology of the eye is also what enables tone mapping algorithms to compress the absolute intensity information of images with little noticeable differences in the final image. By preserving all of the features that are picked up by the low level visual processing cells in the retina most of the intensity information can be discarded. The result of our study to develop a tone mapping algorithm that presented as much visual information to the viewer as possible for a given dynamic range, 8-bits in our case, showed that the automated version of the bilateral filtering algorithm appears to present more information to the viewer than current tone mapping algorithms. However this does not prove that it outputs the maximum amount of information.

In order to say whether the automated algorithm is maximal we need a quantitative measure of how much information is shown to the user. To do this we plan to develop an ideal observer model to estimate how much information is in a tone mapped image in order to maximize the amount of information that it presents to the user. With the completion of this model we can show how close to maximum our automated bilateral filter is and possibly improve it further.

## Figures and Tables

**Figure 11.**
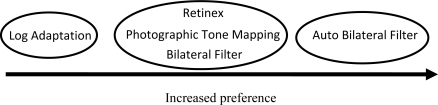
Diagram summarizing the paired comparison results of the second psychophysical experiment. Algorithms are listed from left to right in order of increased preference; in terms of how much visual information or detail seemed to be visible by test subjects.

**Table 1.** Paired Comparison Results, z-statistics and p-values collapsed across participants and scenes.

	**(1) Retinex**	**(2) Histogram Adjustment**	**(3) Photographic tone mapping**	**(4) Bilateral Filter**	**(5). Log Adaptation**	**Z-stat**	**P-Value**
	
1 *vs.* 2	204	96	0	0	0	6.235383	4.51E-10
1 *vs.* 3	152	0	148	0	0	0.23094	0.817361
1 *vs.* 4	158	0	0	142	0	0.92376	0.355611
1 *vs.* 5	193	0	0	0	107	4.965212	6.86E-07
2 *vs.* 3	0	102	198	0	0	−5.54256	2.98E-08
2 *vs.* 4	0	91	0	209	0	−6.81273	9.58E-12
2 *vs.* 5	0	122	0	0	178	−3.23316	0.001224
3 *vs.* 4	0	0	150	150	0	0	1
3 *vs.* 5	0	0	204	0	96	6.235383	4.51E-10
4 *vs.* 5	0	0	0	198	102	5.542563	2.98E-08

**Table 2.** Experiment 2 Paired Comparison Results, z-statistics and p-values collapsed across participants and scenes.

	**(1) Retinex**	**(2) Log Adaption**	**(3) Photographic tone mapping**	**(4) Bilateral Filter**	**(5) Automated Bilateral Filter**	**Z-stat**	**P-Value**
**1 *vs.* 2**	166	134	0	0	0	1.847521	0.064672
**1 *vs.* 3**	134	0	166	0	0	−1.84752	0.064672
**1 *vs.* 4**	114	0	0	186	0	−4.15692	3.23E-05
**1 *vs.* 5**	80	0	0	0	220	−8.0829	6.66E-16
**2 *vs.* 3**	0	108	192	0	0	−4.84974	1.24E-06
**2 *vs.* 4**	0	138	0	162	0	−1.38564	0.165857
**2 *vs.* 5**	0	81	0	0	219	−7.96743	1.55E-15
**3 *vs.* 4**	0	0	148	152	0	−0.23094	0.817361
**3 *vs.* 5**	0	0	110	0	190	−4.6188	3.86E-06
**4 *vs.* 5**	0	0	0	83	217	−7.73649	1.02E-14
